# Concurrent preoperative chemotherapy and three-dimensional conformal radiotherapy followed by surgery for oral squamous cell carcinoma: a retrospective analysis of 104 cases

**DOI:** 10.18632/oncotarget.17363

**Published:** 2017-04-21

**Authors:** Lina Fan, Xuegang Hu, Shihan Lin, Wentu Zhou, Sheng Fu, Hongbing Lv

**Affiliations:** ^1^ Department of Oral and Maxillofacial Surgery, Fuzhou General Hospital of Nanjing Command, PLA, Fuzhou, China; ^2^ School and Hospital of Stomatology, Fujian Medical University, Fuzhou, China

**Keywords:** oral squamous cell carcinoma, three-dimensional conformal radiotherapy, concurrent preoperative chemotherapy, paclitaxel and nedaplatin

## Abstract

**Objectives:**

The objectives of this study were to assess the clinical effects of an integrated program consisting of concurrent preoperative combined paclitaxel and nedaplatin chemotherapy and three-dimensional conformal radiotherapy followed by surgery intended to cure oral squamous cell carcinoma and to determine whether this integrated program is feasible and effective with respect to the treatment of oral squamous cell carcinoma.

**Methods:**

A total of 104 biopsy-confirmed patients who presented with oral squamous cell carcinoma for the first time were included in this study. Concurrent preoperative combined paclitaxel and nedaplatin chemotherapy and three-dimensional conformal radiotherapy were administered to these patients. The most common treatment regimen consisted of infusions of paclitaxel (135-175 mg/m^2^/day), infusions of nedaplatin (150 mg; 80-100 mg/m^2^/day), and irradiation at doses ranging from 1.5 Gy twice daily to 30-40 Gy over 3-4 weeks. The clinical variables evaluated herein included the local recurrence rate, distant metastasis rate, postoperative survival rate, and degree of mouth opening restriction.

**Results:**

The median follow-up time for surviving patients was 60 months, and the median time to progression for all patients was 57.69 months (95% confidence interval, 56.09 to 59.29 months, and the 3-year disease-free survival probability was 97.11%). The effectiveness rate of the integrated program was 98.08%, and the surgery resection rate was 100%. Only a few postoperative adverse reactions were observed. The local recurrence and distant metastasis rates were 1.92% (2 patients) and 2.88% (3 patients), respectively. The titanium rejection and infection reaction rate that led to restriction of mouth opening was only 2.88% (3 patients). Finally, the 5-year post-surgery survival rate was 91.35% (95 patients).

**Conclusion:**

Preoperative combined paclitaxel and nedaplatin chemotherapy and three-dimensional conformal radiotherapy have significant clinical effects leading to positive anti-tumor results in patients with oral squamous cell carcinoma. These treatments also increase the likelihood that patients will undergo successful surgical treatment. The integrated program described herein can increase long-term survival and surgery resection rates and is associated with only a limited number of adverse reactions.

## INTRODUCTION

Over the last ten years, oral squamous cell carcinoma (OSCC) has been the 10^th^ most common malignant tumor. Over 500,000 patients die of OSCC every year [1, 2]. The incidence of OSCC has increased rapidly as a result of exposure to carcinogens, such as betel quid, chewing tobacco, and tobacco smoke, and consumption of alcohol [3]. However, distant metastases are always ignored when patients first present with the disease. Radical ablative surgery followed by chemotherapy or radiochemotherapy is frequently used to treat OSCC. These treatments have decreased local recurrence rates but have not improved 5-year survival rates, as these rates remain lower than 40%. Un-resectable carcinoma treated with radiotherapy alone has a 5-year survival rate of approximately 20% [4, 5]. Previous studies have reported that preoperative treatment with chemotherapy and radiotherapy offers patients many great advantages, as it shrinks tumors, optimizes conditions for surgery, reduces recurrence and metastasis rates, and prolongs patient survival. However, studies attempting to identify more effective and better tolerated novel drugs and treatment regimens are ongoing [6].

It has been demonstrated that paclitaxel, which is isolated from the bark of the yew tree Taxus brevifolia, shows potent antitumor activity in many experimental tumor models. Paclitaxel can induce tumor cells to enter and remain in the G2/M phase and can improve radiation sensitivity [7-9]. The combination of paclitaxel and nedaplatin induction chemotherapy can increase tumor radiosensitivity and thus improve treatment efficiency and increase the likelihood of achieving remission. Paclitaxel is also used in the treatment of esophageal and colorectal cancer. Moreover, paclitaxel has been found to have significant clinical effects in advanced ovarian, breast, non-small cell lung, and head-and-neck cancers, and its use in patients with these diseases has led to optimal outcomes with respect to local disease control, as well as excellent clinical response rates [9, 10].

It has been demonstrated that three-dimensional conformal radiotherapy (3D-CRT) can improve dose distributions, thereby facilitating significant increases in target doses and decreases in lung and heart doses [11]. In virtual simulations, 3D-CRT has been shown to deliver enhanced radiation beams that fit their targets. 3D-CRT can also downstage primary tumors, increase resectability rates, eliminate micrometastases, shrink tumors, improve conditions for surgery, reduce recurrence and metastasis rates, and prolong patient survival [6, 12].

Theoretically, concurrent preoperative combined paclitaxel and nedaplatin chemotherapy and 3D-CRT has many advantages, as this therapeutic regimen downstages primary tumors, increases resectability rates, and eliminates micrometastases. Moreover, the protocol featuring a radiation dose of 40 Gy may have some therapeutic and prognostic advantages over other treatment protocols [9, 11-13].

In this study, we reported the results for a total of 104 patients with biopsy-confirmed OSCC who were treated in our hospital from 1997 to 2009. These patients were treated preoperatively with pre-inductive paclitaxel and nedaplatin chemotherapy with concurrent radiotherapy (40 Gy), followed by selective surgery to remove the residual tumor. The data show that this integrated program is effective as a short- and long-term treatment for OSCC.

## PATIENTS AND METHODS

### Case selection

A total of 104 patients with biopsy-confirmed cases of OSCC who were treated in the Department of Oral and Maxillofacial Surgery, Fuzhou General Hospital of Nanjing Military Command, from 1997 to 2009 were enrolled in this study. The locations, histological differentiations and clinical stages of the patients’ diseases are shown in Table 1.

**Table 1:** Clinical characteristics of the 104 patients

Clinical data	Number of patients	Proportion(%)
**Diseased parts**		
Tongue	42	40.38
Cheek	23	22.12
Gingiva	28	26.92
Mouth floor	11	10.58
**Differentiated**		
Well-differentiated	49	47.12
Moderately differentiated	32	30.77
Poorly differentiated	20	19.23
Undifferentiated	3	2.88
**Clinical stage**		
T2	51	49.04
T3	38	36.54
T4	15	14.42
N1	47	45.19
N2	40	38.46
N3	17	16.35

Pretreatment staging was performed with clinical examinations, ultrasonography, computed tomography (CT) and/or magnetic resonance imaging (MRI) of the head-and-neck region, as well as chest radiography and bone scintigraphy. Additional staging for distant metastases was performed if clinically indicated.

### Case selection criteria

1. All patients enrolled in this study were diagnosed with primary OSCC by pathological examination. Patients with a history of systemic chemotherapy or radiotherapy, concomitant malignancy, active inflammatory bowel disease, active gastric/duodenal ulcers, active infection, severe heart disease, mental disorders, or other severe concurrent diseases were excluded from the study. Pregnant or lactating women were also excluded;
2. Patients with tumors that were too large to cure radically by simple surgery and patients with large lesions that could not be repaired or affected head-and-neck function after surgery were included in the study;
3. Patients without distant metastasis, as determined *via* the above imaging examinations, were included in the study;
4. Patients without contraindications to treatment were included in the study;
5. Patients with a pretreatment Eastern Cooperative Oncology Group performance status of 0 or 1, a life expectancy ≥3 months, and adequate organ function (a leukocyte count of 4,000/mm^3^, a platelet count ≥100,000/mm^3^, a hemoglobin ≥9.0 g/dl, an aspartate aminotransferase (AST) ≤2 times the upper limit of normal (UNL), an alanine aminotransferase (ALT) ≤2 times the UNL, an alkaline phosphatase (ALP) ≤2 times the UNL, a serum bilirubin ≤1.5 mg/dl, and a serum creatinine ≤ the UNL) were included in the study.

All study protocols were approved by the Fuzhou General Hospital Institutional Review Board at each participating center. All patients provided written informed consent before enrolling in this study.

### Clinical stage

The patients were staged according to the 2002 UICC (International Union Against Cancer) staging criteria. Clinical nodal staging was based on neck palpation findings, ultrasonography results, CT or MRI results, post-surgery pathological findings, and the occurrence of mass regression of the tumor after chemotherapy and radiotherapy (tumor size and location were recorded before chemotherapy and radiotherapy). Patients with distant metastases were excluded from the study. The final clinical staging distribution for the study population was as follows: T2 (*n* = 51), T3 (*n* = 38), T4 (*n* = 15), N1 (*n* = 47), N2 (*n* = 40), and N3 (*n* = 17) (Table 1).

### Chemotherapy

The following treatment protocol (administered in the inpatient setting) was used in the study:

The day before chemotherapy, decameth (20 mg) was administered orally, and metoclopramide hydrochloride (10 mg) was administered *via* intramuscular injection.

Day 1: Dexamethasone sodium phosphate (20 mg) was administered orally, metoclopramide hydrochloride (20 mg) was administered *via* intramuscular injection, and 0.9% sodium chloride (500 ml) plus paclitaxel liposome (180 mg) was administered *via* injection.

Day 2: Metoclopramide hydrochloride (10 mg) was administered *via* intramuscular injection, and 0.9% sodium chloride (500 ml) plus nedaplatin (150 mg) was administered by injection.

If the patient did not develop febrile or prolonged neutropenia, prophylactic granulocyte colony-stimulating factor was administered routinely. Patients were monitored by physical examination, and all occurrences of chemotherapy-induced toxicity were recorded weekly throughout the entire treatment period. Toxicity was graded according to the NCI Common Toxicity Criteria 3.0. Blood tests intended to obtain information regarding complete blood counts, serum chemistry and liver function tests were performed at least weekly.^.^

## 3D-CRT

Dental extractions were performed if necessary before 3D-CRT initiation.

Patients were placed in the supine position, immobilized by a thermoplastic mask, and treated with three-dimensional techniques, in accordance with the treatment plan. A total dose of 40 Gy/20-25F/4-5w was delivered daily 5 days per week.

### Surgery

Surgery was performed for patients with residual disease at the primary site or disease involving the neck after chemoradiotherapy [2]. The conventional surgical margins of each tumor were marked in the majority of patients enrolled in the study. Extended resection of the oral carcinoma (which was performed based on the results of pre-chemotherapy and radiotherapy assessments of tumor size and location) combined with partial amputation of the jaw bones and immediate reconstruction of the jaw bones was performed with titanium plates. In this procedure, the submandibular and sublingual glands were excised, and a prosthesis was placed with prosthodontics film. Functional neck dissection was also performed. Even in cases in which an overt residual tumor was absent, selective neck dissection was recommended for patients presenting with N2 or N3 disease after the patients had completed chemoradiotherapy. Surgical reconstruction was performed using locoregional flaps or microsurgical flaps. Frozen tissue sections were collected during surgery to ensure that radical resection was successful.

After completion of therapy, the patients were followed-up at regular intervals. The mean follow-up time was 57 months. Patients underwent physical examinations, radiographic imaging studies, and endoscopy with biopsy of the primary site at 4 to 12 weeks post-therapy. Careful clinical evaluations were combined with regular ultrasound examinations. CT and/or MRI were performed at the discretion of the attending physician. Lesions suspicious for tumor recurrence or additional primary carcinomas were biopsied.

### Quality of life

The Functional Assessment of Cancer Therapy Scale, the Performance Status Scale for Head and Neck Cancer, and the McMaster Head and Neck Radiotherapy Questionnaire, which contained the same standard battery of validated questions as assessments used in previous trials, were utilized prospectively to evaluate quality of life when available [14]. Patients were assessed at 3-month intervals during the first 12 months post-treatment and then annually thereafter.

### Toxicity

Peak acute and late toxicity were defined according to National Cancer Institute Common Terminology Criteria for Adverse Events Version 4.0 and as determined by a retrospective chart review (10). Acute toxicity was defined as toxicity occurring within 90 days of completion of treatment, and late toxicity was defined as toxicity occurring at any time point later than 3 months after completion of treatment (e.g., whether at 3 months or 6 months post-treatment). In locally recurrent disease, radiation-related local late toxicities were not diagnosed, as such toxicities could not be distinguished from tumor progression.

### Follow-up and analysis of failure

Patient survival and tumor outcomes were recorded prospectively. Patients were followed up in the Radiation Oncology Outpatient Clinic at 3, 6 and 12 months post-treatment during the first year after surgery and then followed up annually thereafter. Imaging was performed in patients with suspected disease recurrence so that such patients could undergo a subsequent clinical examination, and histological analysis was performed to confirm all diagnoses of recurrence.

## RESULTS

The median follow-up time was 57 months for all patients and 60 months for surviving patients. The effectiveness rate of preoperative combined paclitaxel and nedaplatin chemotherapy and radiotherapy as a treatment for OSCC was 98.08% (complete response (CR), *n* = 41; partial response (PR), *n* = 61; non-response (NR) *n* = 2). All 104 patients completed preoperative combined paclitaxel and nedaplatin chemotherapy and 3D-CRT, according to the original treatment plan, and suffered from fewer complications, toxicities and side effects than patients treated with other regimens in previous studies. No patients discontinued treatment due to serious complications, and no clinical or disease variables were found to have a significant influence on locoregional control.

### Chemotherapy poisoning and side effects (according to the WHO classification)

All 104 patients completed preoperative chemotherapy. Of these patients, 42 had gastrointestinal reactions of different severities. For example, some patients had mild reactions characterized by nausea and vomiting within 1-2 d after paclitaxel injection that resolved without treatment. Additionally, 7 patients developed fevers, and 12 patients suffered hair loss. Additional patients developed subcutaneous nodules and experienced pigmentation changes and other reactions. No patients discontinued treatment due to renal toxicity, bone marrow growth arrest or pulmonary fibrosis (Table 2). All hematological toxicities experienced by the patients enrolled in the study were mild and reversible. The radiotherapy complications experienced by the patients are shown in Table 3.

**Table 2:** Chemotherapy toxicity and side effects of the 104 patients

Toxic reaction	Toxicity grading					Toxicity incidence(%)
	0	Ⅰ	Ⅱ	Ⅲ	Ⅳ	
**Hematopoietic system**						
HB	100	2	2	-	-	3.85
WBC	79	12	9	2	2	24.04
PLT	85	6	9	4	-	18.27
**Gastrointestinal tract**						
Nausea and vomiting	82	8	6	5	3	21.15
Diarrhea	98	2	4	-	-	5.77
Stomatitis	96	5	3	-	-	7.69
**Renal**						
CR	103	1	-	-	-	0.96
BUN	101	3	-	-	-	2.88
**Liver**						
ALT	99	3	2	-	-	4.81
**Alopecia**	47	26	17	12	2	54.81
**Fever**	89	8	4	3	-	14.42
**Peripheral neuritis**	100	3	1	-	-	3.85

**Table 3:** Radiotherapy complications of 104 patients

Complications	Complications grading					Complications incidence(%)
	0	Ⅰ	Ⅱ	Ⅲ	Ⅳ	
**Oral local reaction**						
Redness, swelling	45	38	12	9	-	56.73
Ulcers, pain	60	23	6	11	4	42.31
Dry mouth	22	65	17	-	-	78.85
Bacterial, fungal infections	83	12	7	2	-	20.19
Bleeding gums, bad breath	61	22	11	10	-	41.35
**Gastrointestinal tract**						
Nausea and vomiting	89	13	2	-	-	14.42
Loss of appetite	51	25	13	10	5	50.96
**Dermal reaction**						
Swelling and itching	20	46	34	4	-	80.77
Damage, ulceration	45	21	33	5	-	56.73
Desquamation	10	43	26	23	2	90.38
Dry	18	32	23	31	-	82.69
Complexion blacken, darken, pigmentary cirrhosis	31	38	26	9	-	70.19
Necrosis	98	3	3	-	-	5.77
**Psychic reaction**						
Anxiety, fear	97	2	4	1	-	6.73
**Limitation of mouth opening**	101	2	1	-	-	2.88
**Radioactive osteomyelitis**	97	3	4	-	-	6.73
**Masticatory function**	102	1	1	-	-	1.92

### Evaluation of the curative effects of chemotherapy and radiotherapy

The effects of the therapy protocol were assessed after every two cycles of therapy, according to the following international standard: a complete response (CR) was defined as the complete clinical and radiographic disappearance of the tumor without the appearance of new lesions, and a partial response (PR) was defined as a reduction in the longest perpendicular diameter of the tumor of at least 50%. A PR also required that no lesions had grown or that no new lesions had appeared for at least 28 consecutive days. A non-response (NR) was defined as a reduction in tumor size of less than 50%. Table 4 shows a summary of the patient responses to the neoadjuvant radiochemotherapy administered herein, according to the abovementioned pre-therapeutic staging classification. The curative effects of the therapy were evaluated four weeks after the end of chemotherapy and radiotherapy (Table 4).

**Table 4:** Clinical characteristics and curative effect of the 104 patients

Clinical date	Curative effect			CR+PR(%)
	CR	PR	NR	
**Diseased parts**				
Tongue	18	23	1	97.62
Cheek	10	13	-	100
Gingiva	11	17	-	100
Mouth floor	2	8	1	90.91
**Differentiated**				
Well-differentiated	25	24	-	100
Moderately differentiated	13	19	-	100
Poorly differentiated	3	16	1	95
Undifferentiated	0	2	1	50
**Clinical stage**				
T_2_	20	31	-	100
T_3_	16	22	-	100
T_4_	5	8	2	86.67
N_1_	24	23	-	100
N_2_	16	24	-	100
N_3_	1	14	2	88.24

### Evaluation of the curative effects of surgery

Eleven patients were originally diagnosed with inoperable disease but regained their eligibility for surgery after receiving concurrent preoperative combined paclitaxel and nedaplatin chemotherapy and 3D-CRT. The rate of post-treatment surgical resection was 100%. This integrated program had fewer post-operative adverse reactions than programs used in other studies.

### Statistical analysis and prospective efficacy

Survival time was defined as the period extending from the first day of treatment until the date of death or the last patient contact. Kaplan-Meier analysis was used to estimate the probabilities of locoregional control, distant metastases-free survival, disease-free survival, and overall survival. The following clinical and disease variables were considered potential outcome predictors in univariate analysis: T stage, lymph node metastasis, radiation dose, age, gender, and chemoradiotherapy regimen. The frequencies of all events were determined by following patients from the date of preoperative chemoradiotherapy initiation to the date of the last clinic follow-up or the date of death, which was determined by reviewing the Social Security Death Index.

**Figure 1:**
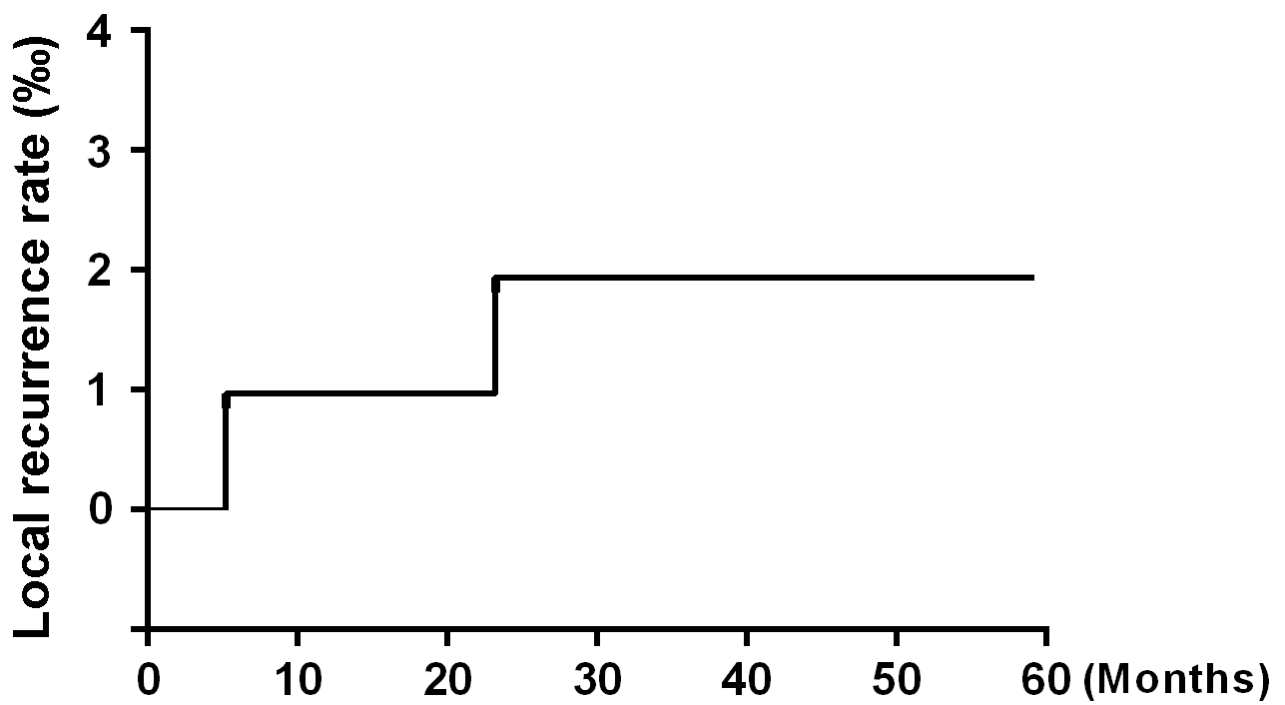
Local recurrence rate

**Figure 2:**
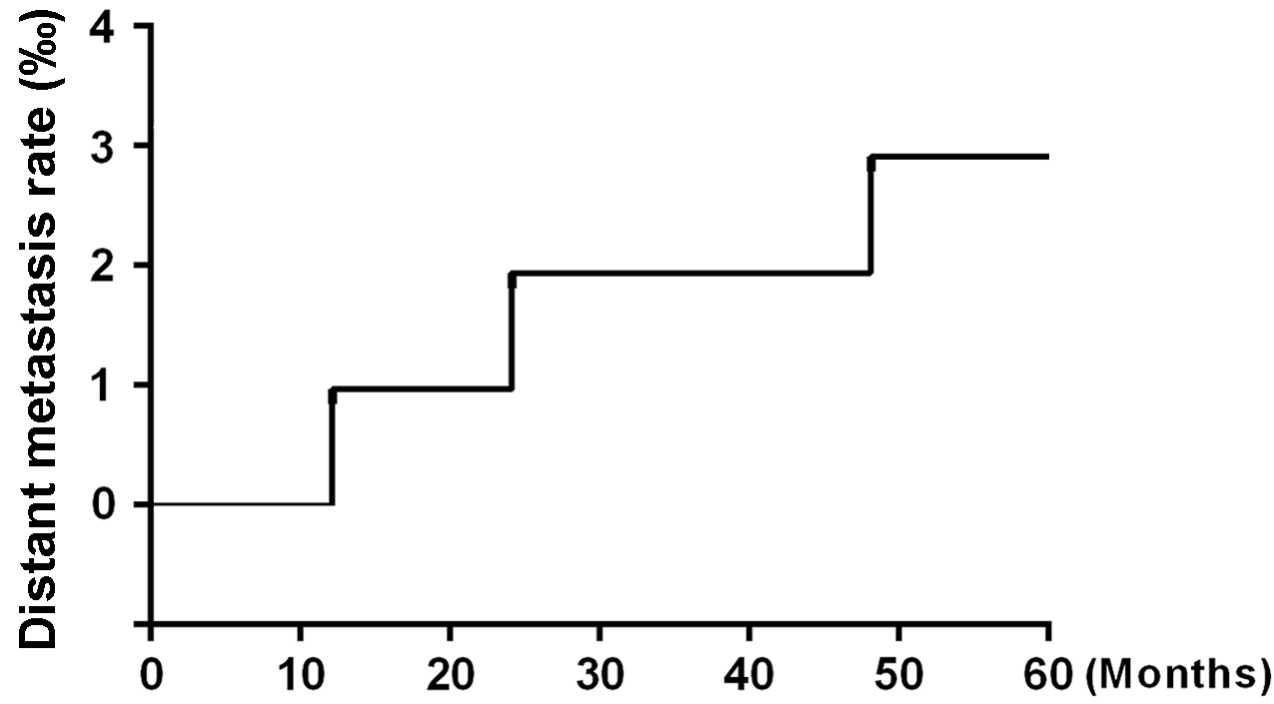
Distant metastasis rate

**Figure 3:**
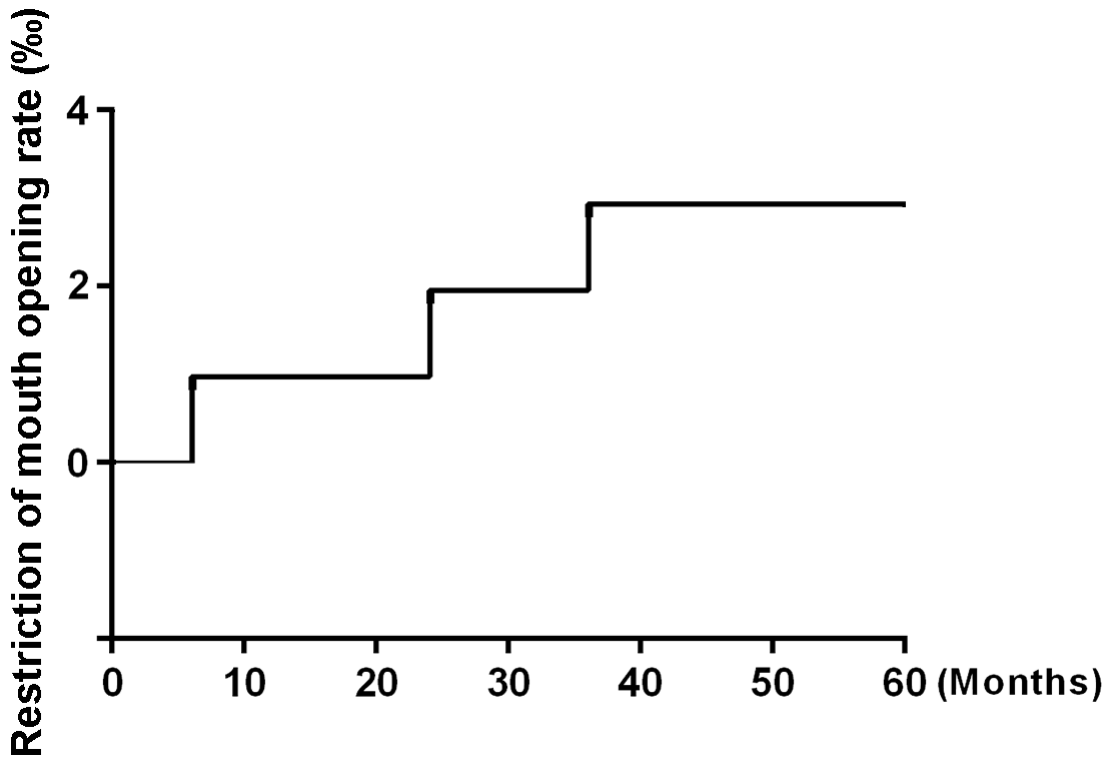
Restriction of mouth opening

**Figure 4:**
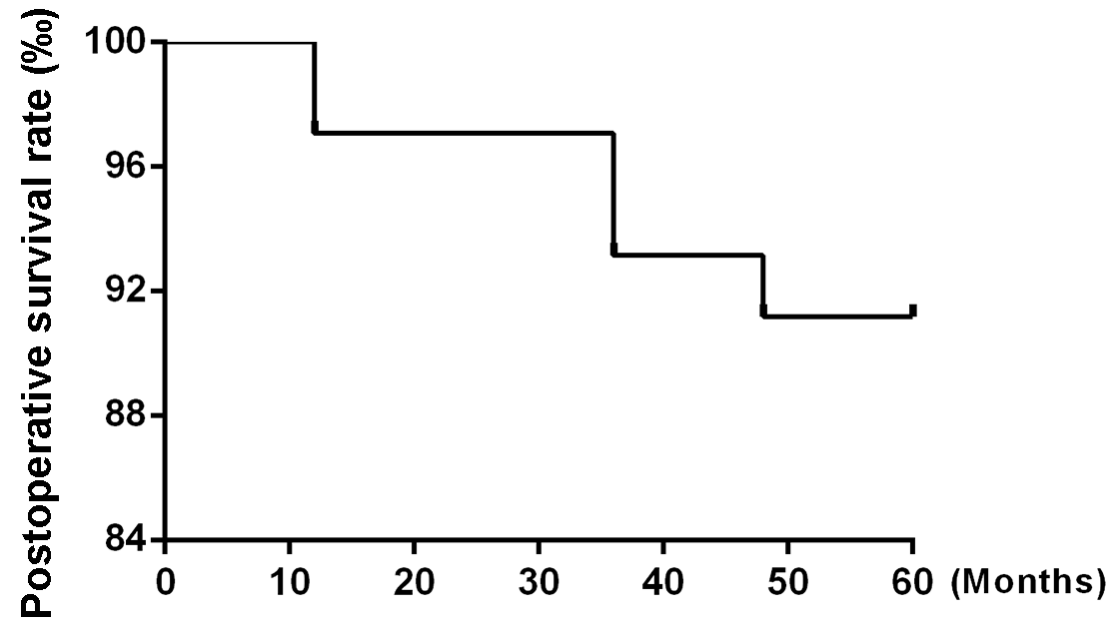
Postoperative survival rate

After 5 years of follow-up, five of the 104 patients enrolled in the study had died, and four patients had been lost to follow-up and were counted as deaths. Ninety-five patients were alive at the end of the observation period. The local recurrence rate was only 1.92% (2/104) (Figure 1), and the distant metastasis rate was only 2.88% (3/104) (Figure 2). The rate of titanium rejection and infection reaction leading to restriction of mouth opening was only 2.88% (3/104) (Figure 3). The 5-year post-surgery survival rate was 91.35% (95/104) (Figure 4). No patients developed facial deformities after surgery. The most important goal of the treatment regimen was to improve patient quality of life after surgery by restoring important patient functions, such as speech, eating, and swallowing.

## DISCUSSION

In the present retrospective analysis comprising more than 100 patients, we reviewed our experience with concurrent preoperative combined paclitaxel and nedaplatin chemotherapy and 3D-CRT followed by surgery for the treatment of OSCC. This integrated program was used to increase locoregional control, improve survival rates, and improve organ preservation [15].

Because of its special anatomical position, the oral and maxillofacial region affects not only related patient aesthetics and physiological functions but also patient mental health [1, 16]. Therefore, treating oral and maxillofacial squamous cell carcinoma is more difficult than treating other cancers. In addition to the above difficulties, clinicians also face the following additional major clinical problem: locoregionally recurrent squamous cell carcinoma. Despite aggressive treatment with surgery and/or radiation therapy, 30-60% of patients with such tumors still eventually develop recurrence or die from their disease [3, 17].

A study evaluated the post-curative treatment rates of locoregional recurrence associated with hyperfractioned radiation therapy, postoperative chemotherapy, or preoperative or postoperative multitechnique radiation therapy. The average rate of tumor recurrence associated with the above treatments was approximately 42% (range, 16% to 75%) at 2 to 5 years post-treatment [6]. Glicksman et al. [18] reported on the utility and superiority of concurrent chemoradiotherapy for advanced head-and-neck cancer. Their results showed that in 101 patients with potentially operable squamous cell carcinoma of the head and neck who received concurrent cisplatin-based chemotherapy and radiotherapy with or without surgery, 49% of patients survived disease free for up to 9 years post-treatment. These patients were followed up for a median of 41 months, and the progression-free survival rate was 78%. Additionally, 22 of the 101 (21.8%) patients enrolled in the study had tumor recurrence. The current methods used for the treatment of OSCC still present many problems, including significant toxicity and side effects. Moreover, these treatments are associated with high recurrence and poor survival rates. In contrast to the above results, our results showed that the overall response rate of primary tumors to chemoradiotherapy was 100% and that the 5-year overall survival rate was 91.35% (95/104) for all the patients enrolled in the study. These patients were followed up for a median of 57 months. Only 2 (1.92%) of 104 patients developed locally recurrent tumors after preoperative combined paclitaxel and nedaplatin chemotherapy and 3D-CRT followed by surgery. Our results also demonstrated that the preoperative regimen used in this study was well tolerated and enabled patients to avoid experiencing serious complications, as the regimen was associated with low toxicity, and no toxicity-related deaths occurred.

In recent studies, the incidence of distant metastases has ranged from 11 to 55% [5, 19]. However, locoregional recurrence was uncommon in our study, as the 5-year local recurrence rate was 1.92% (2/104). Distant metastasis was also infrequent, as the 5-year distant metastasis rate was 2.88% (3/104). Patients were evaluated regarding the occurrence of distant metastases at 12, 24, 48 months post-treatment during follow-up. The mechanism underlying the effects of the above chemotherapy regimen involves a complex DNA-related process. Paclitaxel and nedaplatin, cell cycle-specific radiation sensitizers, induce tumor cells to enter and remain in the G2/M phase, thereby significantly improving tumor radiation sensitivity [9]. Unlike conventional anti-cancer drugs, which can arrest tumor cells at this phase, paclitaxel and nedaplatin can prevent built-in microtubule depolymerization in the cell, leading to the formation of spindle microtubules that are not composed of tubulin. This causes cancer cells to stagnate specifically in the G2 and M phases of mitosis. The treatment has consistent effects across different cell lines and can be adapted for any cell line to inhibit cancer cell proliferation [7, 8, 10]. Based on the findings of previous studies, the key to successful cancer treatment involves developing treatments that interfere with the cell cycle and slow the rate of tumor cell proliferation or induce tumor cells to undergo apoptosis [1, 3]. 3D-CRT also plays an important role in OSCC treatment. The main advantage of 3D-CRT is that this treatment conforms to the shape of the tumor, which reduces the radiation-related toxicity to which the surrounding normal tissues are subjected and allows a higher dose of radiation to be delivered to the tumor than conventional techniques [11, 12, 20]. Preoperative chemoradiotherapy delineates the boundaries between tumor tissues and normal tissues more clearly to facilitate complete tumor resection and decrease the recurrence rate.

The chemotherapy poisoning and side effects rates were also reduced in our study. The incidences of nausea and vomiting (16% to 96%), hematologic abnormalities (10% to 47%), and moderate to severe mucositis (4% to 92%) were reported in previous studies [18, 21, 22]. Renal toxicity (incidence, 7% to 39%), phlebitis, hearing loss, and electrolyte imbalances have also been reported previously [3, 17, 23] but were not observed in this study. Only mild reactions, such as nausea and vomiting, occurred in our patients. Postoperative complications were also rare, and no postoperative mortality was observed. For instance, only 7 cases of radioactive osteomyelitis (6.73%) and 17 cases of grade 2 xerostomia (16.35%) were noted. No patients suffered from severe long-term toxicity. The combination of paclitaxel and nedaplatin induction chemotherapy can increase tumor radiosensitivity and tumor remission efficiency before radiotherapy [11]. 3D-CRT causes less damage to normal tissue than other treatments and thus avoids causing the complications induced by many surgical interventions, such as titanium rejection and infectious reactions, which lead to facial deformities. 3D-CRT, which is used in combination with low-dose chemotherapy at a dose of 40 Gy, may have several advantages over other treatments with respect to efficacy and patient prognosis [11, 24, 25]. Limiting the dose to 40 Gy before surgery reduced the overall radiation dose to which the patient was exposed and the organ-specific dose to which the salivary glands were exposed, thereby aiding in the preservation of salivary gland function and minimizing the risk of post-therapeutic xerostomia. Additionally, low-dose monotherapy has less severe side-effects than high-dose platinum-based or even combined chemotherapy protocols [11, 12, 19].

Another key finding of this study was the relatively high survival rate of the study population. Glicksman et al. [18] reported that among patients with squamous cell carcinoma of the head and neck who received concurrent cisplatin-based chemotherapy and radiotherapy, the progression-free survival rate was 78% after 3 years. Over a median follow-up time of 57 months, only 9 of the 104 patients enrolled in this study, including the 4 patients who were lost to follow-up and thus considered dead, died. Curative surgery was performed without incident, namely, without unanticipated morbidity or mortality after chemoradiotherapy. The long-term survival rate of 91.35% noted herein was superior to those noted in previous studies. When paclitaxel and nedaplatin enter the cell, chain complexes form to prevent DNA replication and transcription, thereby activating a signal transduction pathway that induces apoptosis [7, 9]. Platinum can inhibit tumor cell DNA synthesis and mitosis, causing cells to arrest mainly in G/M phase. Damaged cells trapped at this checkpoint cannot be effectively repaired and eventually die. The local vasculature, which remains intact in the vicinity of the tumor, will increase drug effective concentrations and local absorption rates in the tumor chemotherapy area, resulting primary tumor shrinkage, increases in resectability rates and micrometastasis elimination [8-10]. 3D-CRT can also easily cause tumor shrinkage and adhesion lysis and increase resection rates, providing patients who were previously diagnosed with inoperable tumors with opportunities to undergo surgery. 3D-CRT can obstruct small blood vessels and peritumor lymphatic vasculature, thereby significantly reducing the chances of intraoperative iatrogenic spread. Furthermore, the risk of wound healing disorders, which result from decreased vascularization of the pre-irradiated recipient tissue after reconstructive surgery involving free-flap transfers, decreased with radiation doses of 40 Gy compared to radiation doses of more than 60 Gy, which was used in previous studies [11, 12]. In this study, concurrent preoperative combined paclitaxel and nedaplatin chemotherapy and 3D-CRT followed by surgery yielded good overall and disease-free survival rates for the majority of patients and reliably identified those tumors that did not respond to radiochemotherapy and should thus be treated with more aggressive systemic therapy [13].

The success of the treatment used in this study was dependent on many factors. The prevision of complete preoperative chemoradiotherapy, dental care, daily skin and mouth maintenance during chemoradiotherapy, and aggressive nutritional support, as well as the use of consistent operative techniques, contributed to the multidisciplinary success observed herein. Furthermore, the high patient compliance, low toxicity, successful surgery, and high survival rates yielded by this multimodal protocol can be attributed to careful treatment planning and coordination by surgeons, medical oncologists, and radiation oncologists. This integrated program may be the first in which histopathological examination was superior to clinical and radiological assessments with respect to accurately diagnosing a residual tumor.

Concurrent preoperative combined paclitaxel and nedaplatin chemotherapy and 3D-CRT followed by surgery is an effective and efficient treatment for OSCC in the oral cavity, as this regimen yields encouraging postoperative survival rates and disease-free survival rates [3]. This integrated program can increase 5-year survival rates and results in few side effects and complications, indicating that targeted medicine still has promise as a future cure for cancer [17]. Recent studies have shown that oral drugs cure squamous cell carcinoma of the breast at a higher rate than conventional radiation therapy [26, 27]. This topic may be worth evaluating in a future study. Moreover, studies attempting to identify the appropriate combinations of oral drugs for maximizing quality of life in this cohort of patients may be worthwhile future endeavors.

## SUPPLEMENTARY MATERIALS TABLES










