# Supplementary material for: Concurrent preoperative chemotherapy and three-dimensional conformal radiotherapy followed by surgery for oral squamous cell carcinoma: a retrospective analysis of 104 cases
Source: Oncotarget. 2017 Apr 21;8(43):75557–67. doi: 10.18632/oncotarget.17363 (PMC5650445; doi:10.18632/oncotarget.17363)
Supplement: Supplementary file 1 [file oncotarget-08-75557-s001.pdf]

Concurrent preoperative chemotherapy and three-dimensional conformal radiotherapy followed by surgery for oral squamous cell carcinoma: a retrospective analysis of 104 cases

SUPPLEMENTARY MATERIALS

1 Case Selection Criteria
2 Exclusion criteria
3 Clinical characteristics and curative effect of the 104 patients. See Supplementary_Table_1
4 Chemotherapy toxicity and side effects of the 104 patients. See Supplementary_Table_2
5 Radiotherapy complications of 104 patients. See Supplementary_Table_3
6 The follow-up duration, time for local recurrence, distant metastasis and postoperative survival of all 104 cases. See Supplementary_Table_4
7 Kaplan-Meier analysis of the local recurrence rate
8 Kaplan-Meier analysis of distant metastasis rate
9 Kaplan-Meier analysis of restriction of mouth opening
10 Kaplan-Meier analysis of the postoperative survival rate

1 Case Selection Criteria

1.Age: 21 to 88 years old.

2.Sex: both males and females.

3. All patients were diagnosed with primary OSCC by a pathological examination. Patients were excluded if they had a history of systemic chemotherapy or radiotherapy, concomitant malignancy, active inflammatory bowel disease, active gastric/duodenal ulcer, active infection, severe heart disease, mental disorder, or other severe concurrent disease. Pregnant or lactating women were also excluded;

4. The local range of the tumor invasion was too large to cure radically by simple surgery or there was a large wound that could not be repaired and affected the functional reconstruction after surgery;

5. Distant metastasis in imaging examinations was not found in all of the patients;

6. There were no treatment-related contraindications; and

7. Before treatment, patients had an Eastern Cooperative Oncology Group performance status of 0 or 1, a life expectancy ≥3 months, and adequate organ functions (leukocytes 4,000/mm3, platelets ≥100,000/mm3, hemoglobin ≥9.0 g/dl, aspartate aminotransferase (AST) ≤2 times the upper normal limit (UNL), alanine aminotransferase (ALT) ≤2 times the UNL, alkaline phosphatase (ALP) ≤2 times the UNL, serum bilirubin ≤1.5 mg/dl, and serum creatinine ≤UNL).

All study protocols were approved by the Fuzhou General Hospital institutional review board at each participating center. All patients provided written informed consent before entry into this study.

2 Exclusion criteria

• Previous radiotherapy or chemotherapy.

• Can not tolerate the treatment protocol with systematic diseases such as history of severe pulmonary or cardiac diseases. •

Pregnancy (confirmed by serum or urine β-HCG) or lactation period.

Legal incapacity or limited legal capacity.

•Other previous malignancies within 5 years.

•Evidence of distant metastatic disease and other cancers.

7 Kaplan-Meier analysis of the local recurrence rate

Case Processing Summary			
Total N	N of Events	Censored	
		N	Percent
104	2	102	98.1%

Mean[a]			
Estimate	Std. Error	95% Confidence Interval	
		Lower Bound	Upper Bound
59.135	.618	57.923	60.346

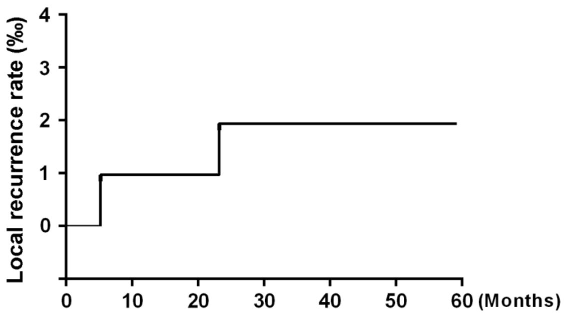

Locoregional recurrence was uncommon. The 5-year local recurrence rate was 1.92% (2/104). A patient with a floor of the mouth cancer failed regionally at 6 months. Another patient with oral tongue cancer demonstrated persistent disease at post-therapy biopsy and succumbed at 24 months. There were no significant clinical or disease variables found to influence locoregional control.

8 Kaplan-Meier analysis of distant metastasis rate

Case Processing Summary			
Total N	N of Events	Censored	
		N	Percent
104	3	101	97.1%

Mean[a]			
Estimate	Std. Error	95% Confidence Interval	
		Lower Bound	Upper Bound
59.077	.581	57.937	60.216

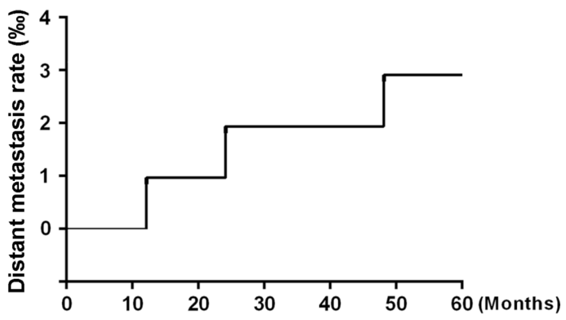

Distant metastasis rate was also uncommon. The 5-year local recurrence rate was 2.88% (3/104). At the 12, 24, and 48 month timepoints, no distant metastases had occurred during follow-up.

9 Kaplan-Meier analysis of restriction of mouth opening

Case Processing Summary			
Total N	N of Events	Censored	
		N	Percent
104	3	101	97.1%

Mean[a]			
Estimate	Std. Error	95% Confidence Interval	
		Lower Bound	Upper Bound
58.904	.657	57.617	60.191

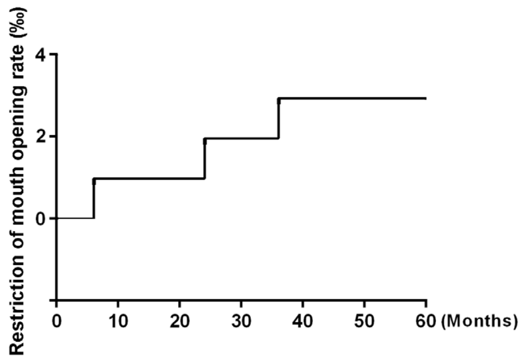

The 5-year restriction of mouth opening was 2.88% (3/104). At the 6, 24, and 36 month timepoints, no restrictions of mouth opening during follow-up had occurred.

10 Kaplan-Meier analysis of the postoperative survival rate

Case Processing Summary			
Total N	N of Events	Censored	
		N	Percent
104	9	95	91.3%

Mean[a]			
Estimate	Std. Error	95% Confidence Interval	
		Lower Bound	Upper Bound
57.462	.904	55.690	59.233

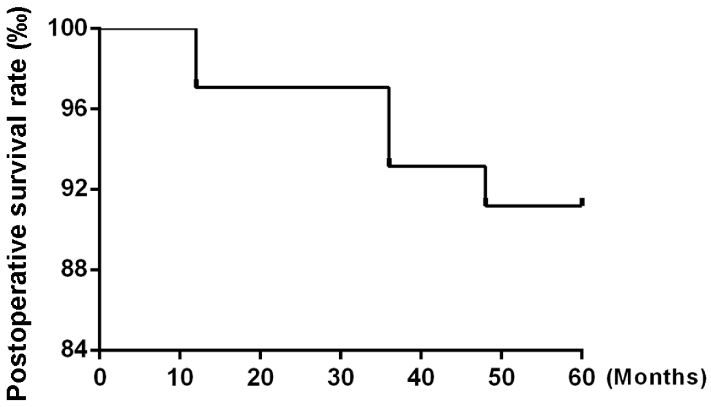

The 5-year postoperative survival rate was 91.35% (95/104). At the 12, 36, and 48 month timepoints, 5 cases had succumbed during follow-up. After the follow-up of the 104 cases of patients, four cases were lost, and these four were counted as deaths.

For Supplementary Tables see in Supplementary Files
